# Supplementary material for: Comparative evaluation of plasma biomarkers of Schistosoma haematobium infection in endemic populations from Burkina Faso
Source: PLoS Negl Trop Dis. 2024 Sep 18;18(9):e0012104. doi: 10.1371/journal.pntd.0012104 (PMC11441675; doi:10.1371/journal.pntd.0012104)
Supplement: S1 Fig — (PDF) [file pntd.0012104.s001.pdf]

**S1 Fig. Venn proportional diagram showing the comparison of CAA and cfDNA testing results.**

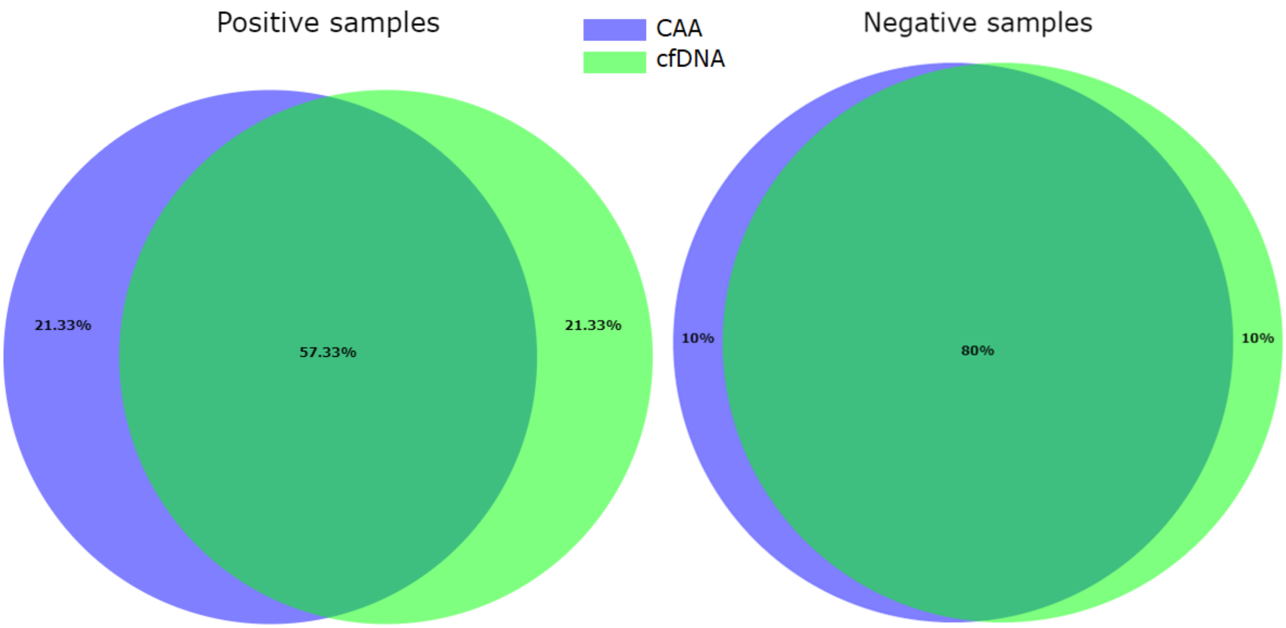


The Venn diagram (DeepVenn, https://arxiv.org/abs/2210.04597) shows the comparison between CAA (blue) and cfDNA (green) testing results. The left panel shows the comparison of positive results, while the right panel shows the comparison of negative results.
