# Supplementary material for: Comparative evaluation of plasma biomarkers of Schistosoma haematobium infection in endemic populations from Burkina Faso
Source: PLoS Negl Trop Dis. 2024 Sep 18;18(9):e0012104. doi: 10.1371/journal.pntd.0012104 (PMC11441675; doi:10.1371/journal.pntd.0012104)
Supplement: S2 Fig — (PDF) [file pntd.0012104.s002.pdf]

S2 Fig. ROC curves illustrating the diagnostic performance of anti-*S.haematobium* antibodies compared to Circulating Anodic Antigen.

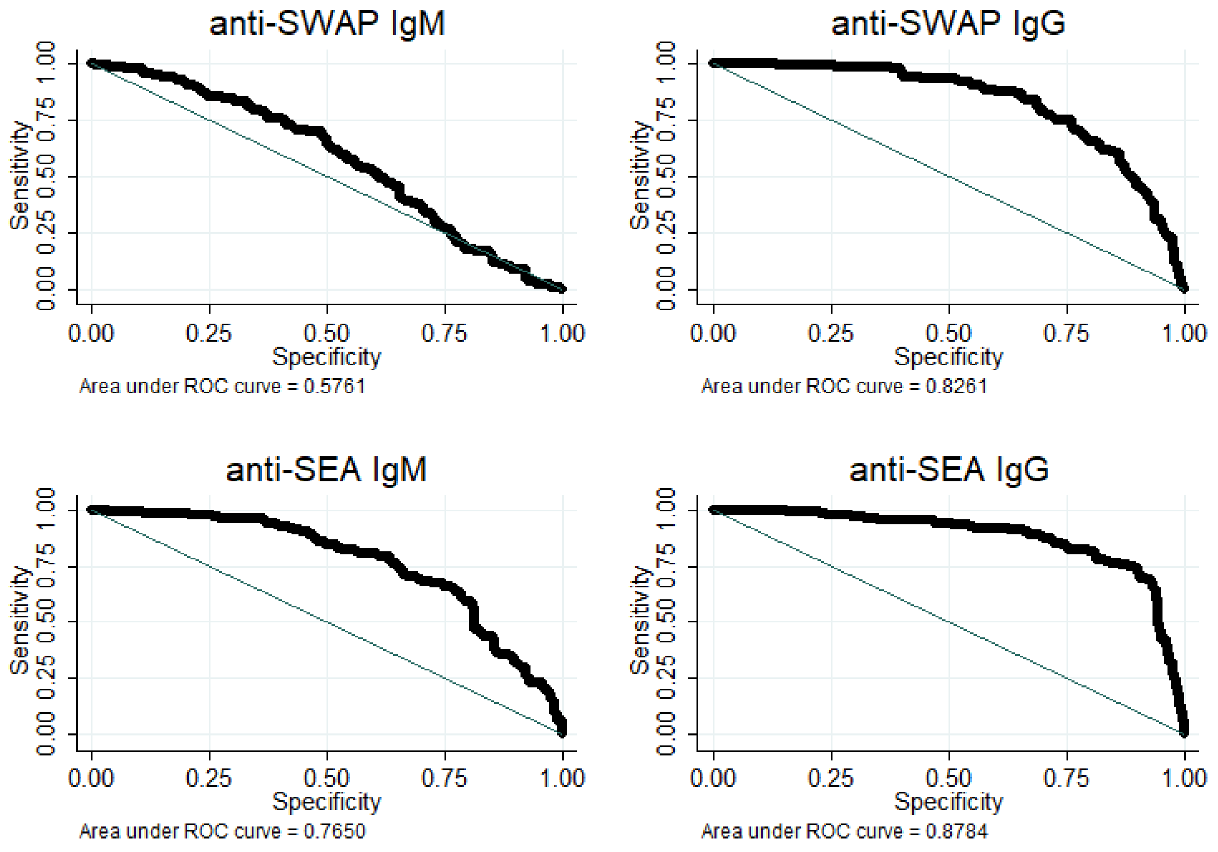

The figure shows the ROC curves illustrating the diagnostic performance of each of the four measured anti-*S.haematobium* antibodies compared to Circulating Anodic Antigen.
