# Supplementary material for: Comparative evaluation of plasma biomarkers of Schistosoma haematobium infection in endemic populations from Burkina Faso
Source: PLoS Negl Trop Dis. 2024 Sep 18;18(9):e0012104. doi: 10.1371/journal.pntd.0012104 (PMC11441675; doi:10.1371/journal.pntd.0012104)
Supplement: Attachment — Submitted filename: Plasma biomarkers manuscript review.docx [file pntd.0012104.s005.docx]

Methods

*Statistical analysis*

Lines 245-250

Query

It is not clear what is “fitted OD, fOD” and how did was determined using the standard curve.

Lines 276-281: Latent Class Analysis (LCA) modelling with two clusters was used to define a binary

(positive/negative) LCA reference [26]. The model included all available manifest variables:

CAA (positive/negative), cfDNA (positive/negative), anti-SWAP IgM level (log10-fOD), anti-

SWAP IgG level (log10-fOD), anti-SEA IgG level (log10-fOD), anti-SEA IgM level (log10-fOD).

The LCA reference therefore defines infection status based on the results of all direct and

indirect biomarker tests.

Query

*The authors clearly described the Composite Reference Standard and one can easily flow the steps; however, the authors can provide more information on the latent class analysis. How was the positive define if at least one of the tests was positive was that sample considered to be positive for S. haematobium. Was a negative result defined by all the tests being negative.*

*Since there is a predefined positive cut off for the ELISA I was suggest using the binary positive/negative instead of the log10-Fod but I stand to be corrected.*

*Line 291*

*The word using was repeated*

*Results*

*Lines 315-316:* *Among subjects who were seropositive for only one specific Ig (12%), the majority was positive for anti-SEA IgG.*

*Query*

*The sentence is not clear please clarify what is meant for only one specific IgG.*
