# Supplementary material for: Comparative evaluation of plasma biomarkers of Schistosoma haematobium infection in endemic populations from Burkina Faso
Source: PLoS Negl Trop Dis. 2024 Sep 18;18(9):e0012104. doi: 10.1371/journal.pntd.0012104 (PMC11441675; doi:10.1371/journal.pntd.0012104)
Supplement: Attachment — Submitted filename: Rebuttal letter_PLOS NTDs.docx [file pntd.0012104.s006.docx]

Pisa, 31^st^ August 2024

Uwem Friday Ekpo

Academic Editor

Eva Clark

Section Editor

PLOS Neglected Tropical Diseases

Dear Dr. Ekbo, dear Dr. Clark,

we are grateful for the review of the manuscript “Comparative evaluation of plasma biomarkers of *Schistosoma haematobium* infection in endemic populations from Burkina Faso” (PNTD-D-24-00453).

We thank the reviewers for their useful comments which helped us to clarify some important issues. We have addressed the reviewers’ concerns in the point-to-point response here below and have edited the manuscript accordingly. We also took the opportunity to correct three mistakes that we recognized after submission: i) CAA level 4 is for antigen concentrations equal or higher than 10000pg/ml, not in the 10000-100000pg/ml range; ii) the colors used to indicate anti-*S.haematobium* antibodies in Figure 4 did not match those in Table 2 and Figure 5; iii) the legend of the three panels of Figure 6 had a little discrepancy.

We hope that the revised manuscript is now suitable for publication in PLOS Neglected Tropical Diseases.

With many thanks for your attention,

yours sincerely,

Prof. Valentina Mangano

**Point-to-point response to reviewers**

**Reviewer#1**

Methods

Lines 245-250: It is not clear what is “fitted OD, fOD” and how did was determined using the standard curve.

Thank you for this observation. On each ELISA plate, a standard curve was fitted to the ODs obtained for the positive control pool dilutions by least squares minimisation using a three parameters sigmoid model and the Solver plugin in Microsoft Excel. To account experimental variability across ELISA plates, the OD of test samples were normalised using the fitted curve parameters on each plate. We have expanded the description of the methodology for better clarity.

Since there is a predefined positive cut off for the ELISA I was suggest using the binary positive/negative instead of the log10-Fod but I stand to be corrected.

Thank you for this comment. We have used binary (positive/negative) ELISA results for the comparison of diagnostic performance with the three references: i) CAA, ii) CRS, iii) LCA. We have also used ELISA results as quantitative variables (log10fOD) to explore whether antibody levels: i) vary according to CAA positivity, ii) correlate with CAA concentration.

Lines 276-281: Latent Class Analysis (LCA) modelling with two clusters was used to define a binary (positive/negative) LCA reference [26]. The model included all available manifest variables: CAA (positive/negative), cfDNA (positive/negative), anti-SWAP IgM level (log10-fOD), anti-SWAP IgG level (log10-fOD), anti-SEA IgG level (log10-fOD), anti-SEA IgM level (log10-fOD). The LCA reference therefore defines infection status based on the results of all direct and indirect biomarker tests. The authors clearly described the Composite Reference Standard and one can easily flow the steps; however, the authors can provide more information on the latent class analysis. How was the positive define if at least one of the tests was positive was that sample considered to be positive for S. haematobium. Was a negative result defined by all the tests being negative.

Thank you for this comment. While the Composite Reference Standard is defined by the authors that use an algorithm to assign positive and negative status to a sample based on the results of the different tests, Latent Class Analysis (LCA) is a statistical procedure that is used to identify unknown groups of individuals in a population based on sharing of characteristics. In our case, LCA was employed to identify *S. haematobium* positive and negative subjects based on all available plasma biomarker data (CAA, cfDNA, specific antibodies). We have now modified the statistical methods for better clarity.

Line 291: The word using was repeated

Thank you, the repetition has been eliminated.

Results

Lines 315-316: Among subjects who were seropositive for only one specific Ig (12%), the majority was positive for anti-SEA IgG. The sentence is not clear please clarify what is meant for only one specific IgG.

Thank you for this observation. For “subjects who were seropositive for only one specific Ig” we meant “subjects who were seropositive for only one of the four measured anti-*S.haematobium* antibodies”. We have modified the sentence for better clarity.

Discussion

One of the challenges in the develop of high sensitive and specific point of care diagnostic tools which can be used low resource endemic areas is the lack of appropriate diagnostic tools. The Composite Reference Standard (CRS) and Latent Class Analysis (LCA) methods described in the manuscript by Mireille Ouedraogo and colleagues can be used as references for new diagnostic tools for NTDs.
Considering that schistosomiasis is mostly endemic in poor resource areas the techniques described in this manuscript will not be applicable in these settings. The techniques described are expensive, they require sophisticated laboratories and trained personnel among other things especially the PCR methods so the is still need to develop tools for use in poor endemic settings.

Thank you for this observation. We agree that none of the methods employed is adequate for point-of-care diagnosis of urogenital schistosomiasis in resource limited settings as they do not comply with ASSURED criteria. We have now included this consideration in the discussion.

**Reviewer #2**

Methods

The objectives of the study are clearly stated. The study design is appropriate. The population is clearly described and appropriate. Correct statistical methods were used. There are no ethical or regulatory requirement concerns. A few suggestions …

The authors should consider providing a power analysis or mention their sample size calculations.

Thank you for the observation. Sample size was not determined by power analysis but by the availability of archived plasma specimens, since this was a retrospective study. We have integrated this explanation in the study design section.

We have calculated that, on a total population of 1769 subjects (census data collected at the time of the study), a sample size of 269 subjects would be needed to obtain a proportion of 29.1% positive subjects (the observed CAA and cfDNA positivity) with a confidence level of 95% that the real value is within ±5% of the measured value. Our sample size was larger than required (n=406). However, we reckon that such post-hoc power analysis would not add useful elements to the interpretation of the results, which can be based on confidence intervals, as pointed out by several authors. Therefore, we did not made integrations to the manuscript in this regard.

Line 257: Please specify the type of confidence intervals used.

We used the 95% Confidence Interval of the proportion (lines 259-60).

Line 258: Did you fit unadjusted and adjusted logistic regression models? Please specify the variables considered and/or used in the final model(s). Did you obtain/report odds ratios? Please clarify.

Thank you for pointing this out. We performed univariate logistic regression to assess the association between categorical/binary testing results. We did not include other variables (e.g. demographic factors) in the models as confounders or effect modifiers, since we reckon this type of analysis is out of the diagnostic scope of the present investigation. Results were expressed reporting Odds Ratio with its 95% Confidence Interval and p-value. We have now integrated the method section to include this information.

Lines 259-262: Why did you use Spearman (for ranked data) as opposed to Pearson (for normal data) to examine correlations with antibody levels and antigen concentration? You then used linear regression to examine antibody levels and antigen concentration, which requires normal data. If these variables were transformed to normalize them, please specify so here. What variables were considered for the linear regression models?

Thank you for pointing this out. The distributions of antibody levels and antigen concentration were normalised by logarithmic transformation. Pearson test has now been performed to assess correlation between the two, instead of Spearman. Univariate linear regression was used to assess association with antibody levels. We have now modified the statistical methods for better clarity, as well as the results.

Results
The results match the analysis plan for the most part. The results are clearly presented. Most tables and figures are appropriate. A few suggestions …

Table 1: Consider including the total N in a table footnote so you can delete the N total column (feels unnecessary to have a whole column for a single number).

Thank you for the suggestions, we have modified the table accordingly.

Lines 322-325: Are these unadjusted or adjusted logistic regressions models? Are there any variables you could consider in adjusted models? If so, a table of the results would be needed.

We used univariate logistic regression models to test the association between categorical/binary testing results. We did not include other variables (e.g. demographic factors) in the models as confounders or effect modifiers, since we reckon this type of analysis is out of the diagnostic scope of the investigation (please see above).

Throughout the paper, P-values not really needed since you report 95% CI, which show the significance.

We agree that p-values are somewhat redundant when reporting 95% CI, but we prefer to keep them for better clarity to all readers.

For all tables with 95% CIs, consider combining the statistic with the corresponding CI so you do not have multiple LCL and UCL column labels. For example, in Table 3, you could use Se (95% CI), Sp (95% CI), Agreement, Cohen’s K as the columns.

Thank you for the suggestions, we have modified the tables accordingly.

Did you actually use any linear regression models? No related results were reported in the text.

We performed univariate linear regression to test the association between CAA positivity and specific antibody levels (lines 408-411).

Discussion

Overall the paper is well written, and the public health relevance is clear. The statistical methods and overall conclusions are appropriate. Addressing some of the gaps mentioned in the methods and results will strengthen the manuscript even more.

Thank you very much.

Minor
Line 87: ‘generation’ should be plural
Line 167: remove ‘)’ before the period

Thank you, we have now corrected these and other typos.
